# Comorbidity Index as a Predictor of Mortality in Pediatric Patients With Solid Tumors

**DOI:** 10.3389/fped.2019.00048

**Published:** 2019-03-01

**Authors:** Luz María Torres-Espíndola, Joel Demetrio-Ríos, Liliana Carmona-Aparicio, César Galván-Díaz, Martín Pérez-García, Juan Luís Chávez-Pacheco, Julio Granados-Montiel, Israel Torres-Ramírez de Arellano, Arnoldo Aquino-Gálvez, Manuel De Jesús Castillejos-López

**Affiliations:** ^1^Pharmacology Laboratory, National Institute of Pediatrics, Mexico City, Mexico; ^2^Neuroscience Laboratory, National Institute of Pediatrics, Mexico City, Mexico; ^3^Oncology Service, National Institute of Pediatrics, Mexico City, Mexico; ^4^Tissue Engineering, Cell Therapy and Regenerative Medicine Unit, National Institute of Rehabilitation, Mexico City, Mexico; ^5^Pathology Department, National Institute of Neurology and Neurosurgery, Mexico City, Mexico; ^6^Biomedical Oncology Laboratory, National Institute of Respiratory Diseases, Mexico City, Mexico; ^7^Epidemiological Surveillance Unit, National Institute of Respiratory Diseases, Mexico City, Mexico

**Keywords:** charlson comorbidity index, solid tumor, pediatric, cancer, chemotherapy, mortality

## Abstract

**Purpose:** The objective of this study was to determine whether a comorbidity index could be used to predict mortality in pediatric patients with chemotherapy-treated solid tumors.

**Methods:** Pediatric patients who underwent chemotherapy treatment for solid tumors were included, and demographic, clinical, and comorbidity data were obtained from patient electronic records.

**Results:** A total of 196 pediatric patients with embryonic solid tumors were included. Metastatic tumors were the most frequently observed (*n* = 103, 52.6%). The most common comorbidities encountered for the Charlson comorbidity index (CCI) were cellulitis (*n* = 24, 12.2%) and acute renal failure (*n* = 15, 7.7%). For the Pediatric Comorbidity Index (PCI), the most frequent comorbidities were pneumonia and sepsis, with *n* = 64 (32.7%) for each. We evaluated established the prognostic values for both indexes using Kaplan-Meier curves, finding that the CCI and PCI could predict mortality with *p* < 0.0001.

**Conclusion:** Using the PCI, we observed 100% survival in patients without comorbidities, 70% survival in patients with a low degree of comorbidity, and 20% survival in patients with a high degree of comorbidity. Greater discrimination of probability of survival could be achieved using degrees of comorbidity on the PCI than using degrees of comorbidity on the CCI. The application of the PCI for assessing the hospitalized pediatric population may be of importance for improving clinical evaluation.

## Introduction

Childhood cancer is a health problem worldwide, and with a reduction of infectious diseases in low- and middle-income countries, this pathology has become the principal cause of mortality for this population (1). Cancer is the second leading cause of death globally and was responsible for 8.8 million deaths in 2015. Globally, nearly 1 in 6 deaths are due to cancer. It is estimated that 70% of deaths from cancer occur in low- and middle-income countries (2).

Mortality in the pediatric population varies considerably according to tumor type, tumor site, nutritional status, patient age, and tumor stage. In adults, there is an effect of gender on mortality; for example, women with pulmonary cancer have a better prognosis than men with the same diagnosis (3, 4), and the underlying reasons are still debated. Several epidemiological studies have established mortality differences according to the socioeconomic status and/or ethnic group (5–8), but just a few have evaluated the effect of any comorbidity on the prognosis of these patients.

Comorbidity is defined as the presence of one or more diseases among patients with the same disease index, and it is measured with the sum of the number of diseases present in one individual through comorbidity scales that combine the number and severity of the concomitant diseases (9). Increased comorbidity scores are associated with (a) an increased risk of hospitalization due to treatment-associated toxicity, (b) increased hospitalization costs and (c) decreased patient quality of life. These effects increase mortality risk (3–7), even without considering impact on the patient's family or treatment cost.

Recently, epidemiological research studies have started to focus on the evaluation of comorbidities in an attempt to measure associations of these comorbidities with treatment outcomes and patient recovery. This research has shown that comorbidities are related to disease prognosis for patients with cancer and may help to predict patient survival (10–14). The Charlson comorbidity index (CCI), which has been validated in adult populations, is the most frequently used comorbidity index in adult populations and overall (15). This method predicts 1-year mortality for a patient based on multiple (22 in total) comorbid conditions, including cardiac disease, AIDS, and cancer (16, 17). Currently, there are no standardized methods for giving an exact morbimortality percentage in a pediatric, which could be used for chronic or lethal pathologies to improve hope and cope with their disease. The frequent practice of excluding patients with common comorbidities and the lack of systemic measurement in studies of pediatric cancer patients limit the evidence for informed decision making; therefore, baseline measurements using a prognostic approach for clinical management and decision making in pediatric populations with comorbidities undergoing chemotherapy treatment remain unclear. The aim of this study was to evaluate if the comorbidity indexes can predict the mortality in pediatric patients with chemotherapy-treated solid tumors.

## Materials and Methods

A retrospective study was conducted by reviewing electronic clinical records of patients diagnosed with the International Classification of Diseases (ICD)-10-compatible solid tumors diagnosis. The study was approved by the Research and Ethics Committees of our institute (National Institute of Pediatrics, NIP) with the registry number 068/2013. One hundred ninety-six pediatric patients (1 month to 17 years old) treated in a tertiary-level hospital (from August 2013 to May 2017) with chemotherapy according to the Mexican Cancer Technical Protocols for Children (18) were analyzed.

### Data Information Source

Data were collected from a survey through the electronic clinical record system and included information regarding demographics, clinical data, and comorbidities presented by the patients at diagnosis, such as acute myocardial infarction, heart failure, peripheral arterial disease, cerebrovascular disease, dementia, chronic respiratory failure, gastroduodenal ulcer, rheumatic disease, hemiplegia, mild chronic hepatopathy, moderate/severe hepatopathy, diabetes without damage to the target organs, diabetes with damage to the target organs, solid tumor, or neoplasia with metastasis, leukemia, and AIDS. Follow-up information was documented during chemotherapy cycles. A database was designed to include living patients and for deceased patients.

### Charlson Comorbidity Index (CCI)

Twenty-three diseases were studied using the CCI; each condition was given a score of 1, 2, 3, or 6 according to the risk of death associated with this condition. The sum of the scores provided a total score that predicts mortality, and is classified as low comorbidity for a score of 0–2 points or high comorbidity for a score ≥ 3 points (17).

### Pediatric Comorbidity Index (PCI)

For the PCI, 30 diseases were evaluated. For the conceptualization of this new index, the relative risks for prediction of 1-year mortality reported for the most common pathologies were used according to Tai et al. (19), and we added some other comorbidities of prognostic importance to the NIP, such as renal disease, lymphoma, HIV/AIDS and associated genetic syndromes. A panel of experts in pediatric oncology (doctors and researchers) from the NIP granted a weighted score of 1, 2, or 4 according to the severity of the comorbidity, assigning a score of 1 for granulocytosis, pneumonia, hydrocephalus, pyrexia, coagulopathy, septicaemia, type 1 diabetes, arrhythmia, mild hepatopathy, pulmonary contusion, obesity, and short size [those with an odds ratio (OR) between 1 and 5]; a score of 2 was assigned for pneumonitis, acidosis, hypertension, malnutrition, cerebrovascular events, traumatic brain injury, candidiasis, renal disease, lymphoma, solid tumor without metastasis, and congenital heart disease (adapted by putting together a ventricular septal defect and a congenital subaortic stenosis encompassing any congenital heart disease), which had an (OR between 6 and 20); and finally a score of 4 was given for heart failure, leukemia, shock state, asphyxia, associated genetic syndromes, HIV/AIDS, solid tumor with metastasis, insipid diabetes and brain cancer because they had an (OR higher than 20) (19).

To further classify the burden of comorbidity, three categories were created according to the scores obtained from the total sum of each patient: 0–2 points (absent), 3–5 points (low) and 6 or more points (high). This index was applied from 0 to 17 years; comorbidities present were recorded at diagnosis and patient admission.

### Statistical Analysis

#### Univariate Analysis

A summary of descriptive statistics was created according to the operationalization of the variables defined in the protocol. Each variable was analyzed separately by means of frequency measurements and percentages for qualitative variables, and measures of central tendency, as well as their corresponding dispersion, were used for quantitative variables.

#### Bivariate Analysis

Results for the CCI and PCI were compared via non-parametric tests of medians using the Mann-Whitney U. A value of *p* < 0.05 was considered statistically significant.

#### Survival Analysis

Kaplan-Meier (KM) curves were plotted to determine existence of differences in survival probability according to the calculated comorbidity category (absent, low, or high) presented by the patients. In the log-rank test, a value of p < 0.05 was considered statistically significant.

#### Cox Regression

Multivariate analysis by Cox's proportional hazards ratio (HR) model was used to test the significance of prognostic factors including gender, age, CCI, PCI, with the relative risks estimated in two models. The HR and 95% confidence intervals (95% CI) of the prognostic factors were calculated, and the results were considered significant if the 95% CI excluded the null value. Data was analyzed using the IBM SPSS Statistics 20 statistical package.

## Results

### General Description of the Cohort

From the 196 patients with embryonic solid tumors included in the study, 78 (39.8%) were female and 118 (60.2%) were male, with a median of 8 years old (range, 3–13 years). Seven types of embryonic solid tumors were analyzed. Medulloblastoma, neuroblastoma, glioblastoma, ependymoma, pineal tumor, neuroepithelial tumor, astrocytoma, and neuroectodermal tumor were grouped into central nervous system (CNS) tumors and were the most frequent (*n* = 53, 27%). Retinoblastomas were the second most common (*n* = 35, 17.8%) followed by soft tissue sarcomas (*n* = 34, 17.3%).

### Comorbidity Frequencies in Patients With Embryonic Solid Tumors

Metastatic tumors were the most frequent for both indexes (*n* = 103, 56.2%). Table 1 shows comorbidities evaluated with both indexes; for CCI, the most frequent were cellulitis (*n* = 24, 12.2%), followed by acute renal failure (*n* = 15, 7.7%) and hypertension (*n* = 15, 7.7%). For PCI, the most frequent comorbidities were pneumonia (*n* = 64, 32.7%), septicaemia (*n* = 64, 32.7%) and malnutrition (*n* = 61, 31.1%).

**Table 1:** Frequency of comorbidity indexes in children treated with chemotherapy (*n* = 196).

**COMORBIDITY INDEXES**					
**Charlson comorbidity index (CCI)**			**Pediatric comorbidity index (PCI)**		
**Comorbidities evaluated**	***N***	**%**	**Comorbidities evaluated**	***N***	**%**
Cellulitis	24	12.2	Pneumonia	64	32.7
Acute renal failure	15	7.7	Septicaemia	64	32.7
Hypertension	15	7.7	Malnutrition	61	31.1
Depression	9	4.6	Shock state	46	23.0
Hemiplegia	6	3.1	Atopy	30	15.3
Heart failure	6	3.1	Convulsive crisis	29	14.8
Ulcerative disease	3	1.5	Brain cancer	22	11.2
Peripheral vascular disease	2	1.0	Hydrocephalus	20	10.2
Cerebrovascular disease	2	1.0	Hypertension	15	7.7
Asthma	1	0.5	Acute renal failure	15	7.7
Mild chronic hepatopathy	1	0.5	Candidiasis	14	7.1
Rheumatic disease	1	0.5	Coagulopathy	7	3.6
Leukemia	1	0.5	Heart failure	6	3.1
Lymphoma	0	0	Obesity	6	3.1
Presence of moderate/severe hepatopathy	0	0	Congenital heart defect	5	2.6
HIV-AIDS	0	0	Arrhythmia	5	2.6
Dementia	0	0	Genetic syndrome	4	2.0
DM without damage to target organ	0	0	Short size	3	1.5
Myocardial infarction	0	0	Traumatic brain injury	2	1.0
DM with damage to target organ	0	0	Diabetes insipidus	2	1.0
Warfarin intake	0	0	Cerebrovascular event	2	1.0

### Mortality Differences Associated to Comorbidity Burden

**Figure 1:**
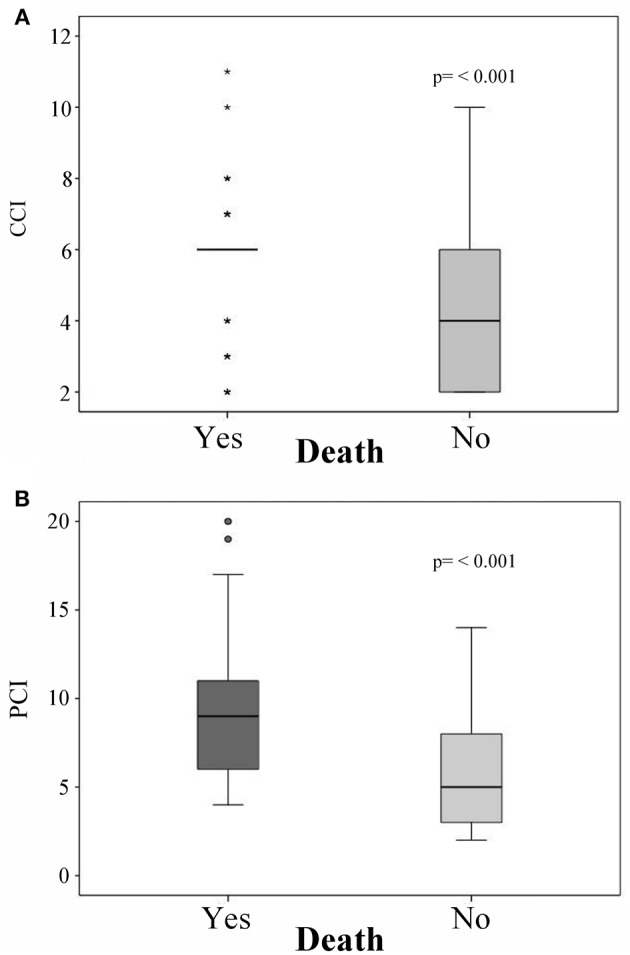
Mortality predictions with the **(A)** Charlson comorbidity index (CCI) and **(B)** Pediatric Comorbidity Index (PCI). ^*^Represents an outlier above the 99th percentile or below the 1th percentile.

Comparison of CCI scores between deceased and living patients, showed significant differences, with median scores of 6 points for deceased patients and 4 points for living patients (*p* < 0.001; Figure 1A); PCI also showed significant differences, with median scores of 9 points for deceased patients and 5 points for living patients (*p* < 0.001; Figure 1B). During follow-up, 70 patients died (35.7%); the most common causes were disease progression (*n* = 23, 11.7%) followed by septic shock (*n* = 14, 7.1%) and cardiorespiratory arrest (*n* = 11, 5.6%).

### Mortality According to the Diagnosis

The highest number of deceased patients was found in the group of CNS tumors (27.57%), followed by retinoblastomas (21.4%), sarcomas (14.2%), osteosarcomas (12.86%), and Ewing sarcomas (10%).

### Comorbidity Indexes as Prognostic Factors for Mortality

**Figure 2:**
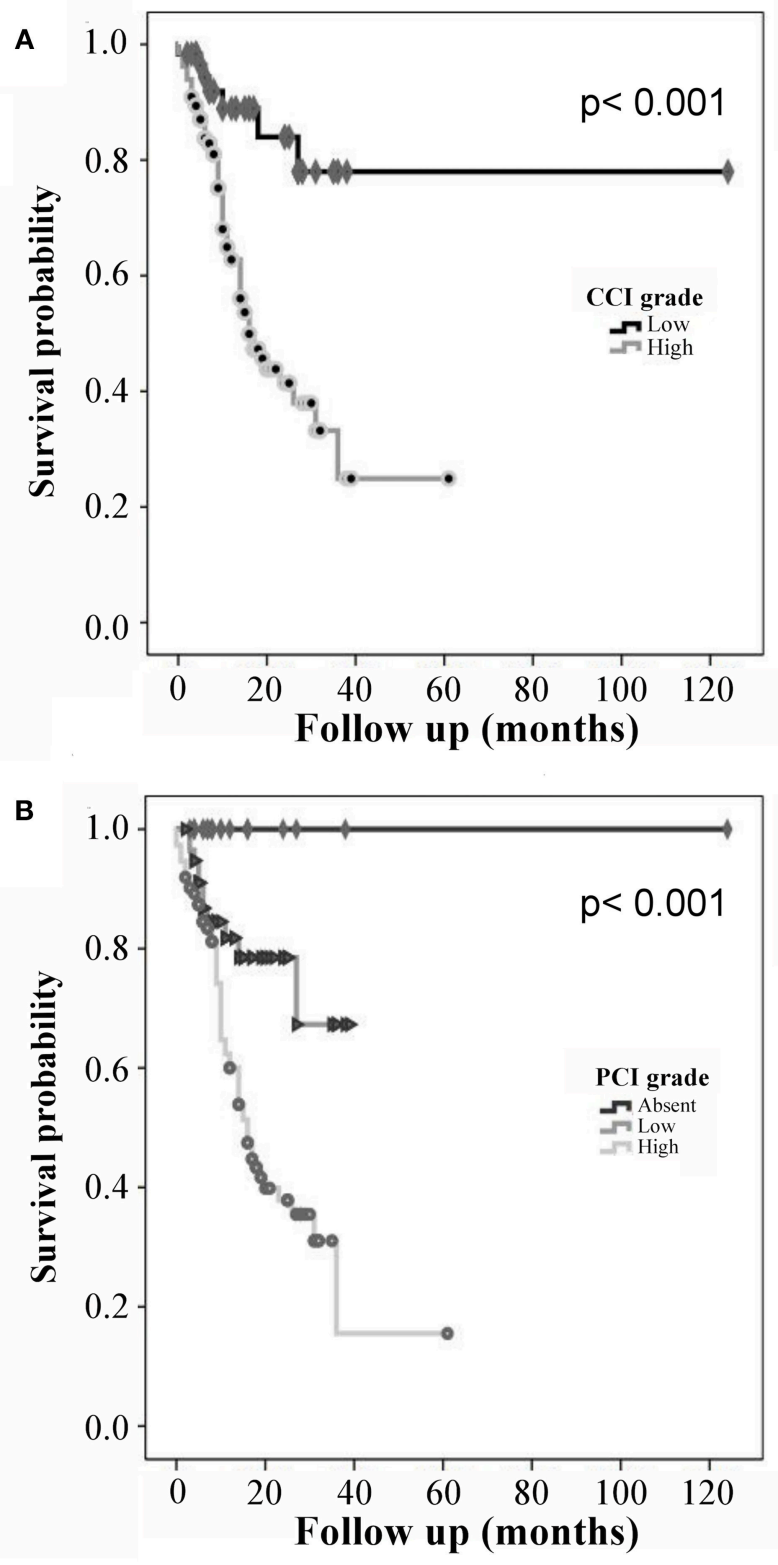
Kaplan-Meier curves for prediction of mortality: **(A)** Charlson comorbidity index (CCI) and **(B)** Pediatric Comorbidity Index (PCI).

Differences in survival were found by using the two comorbidity indexes; 5-year survival for the CCI was 80% for patients with a low comorbidity category and 24% for those with a high comorbidity category. The 5-year survival for the PCI was 100% in the absence of comorbidity, 68% for low comorbidity, and 18% for high comorbidity. When survival curves according to CCI group (high comorbidity = 1 vs. low comorbidity = 2) within the months from diagnosis to the end of follow-up were compared, a statistically significant difference was found (*p* = 0.000066; Figure 2A). Similarly, a comparison of survival curves according to PCI group (high comorbidity = 1 vs. low comorbidity = 2 vs. absent comorbidity = 3) revealed a statistically significant difference with *p* = 0.000044 (Figure 2B).

### Multivariate Cox Regression Models for Mortality

We found an increase in the number of deaths in patients who presented high comorbidity for both indexes. In a Cox survival model adjusted for gender and age, for every additional unit of PCI, the risk of death increased up to 20% (HR: 1.202; 95% CI: 1.078–1.341, *p* < 0.001; Table 2). We also observed a similar mortality risk for each unit added of CCI (HR: 1.152, 95% CI: 1.088–1.220, *p* = 0.001).

**Table 2:** Multivariate Cox regression models for mortality in children treated with chemotherapy.

**Variable**	**Multivariate cox model (CCI)**			**Multivariate cox model (PCI)**		
	**HR**	**95% CI**	***p***	**HR**	**95% CI**	***p***
Age (years)	0.949	0.906–0.995	0.029	0.965	0.921–1.012	0.141
Female gender	1.034	0.633–1.688	0.895	1.030	0.628–1.687	0.908
CCI	1.202	1.078–1.341	0.001			
PCI				1.152	1.088–1.220	< 0.001

*HR, hazard ratio; CI, confidence interval; CCI, charlson comorbidity index; PCI, pediatric comorbidity Index*.

## Discussion

This study found that high comorbidity burden acts as a mortality prognostic factor in a cohort of pediatric patients with solid tumors. To avoid possible interference or confusion from other factors, we chose patients who were treated with only chemotherapy. Interestingly, the results showed that the highest scores for both comorbidity indexes (PCI ≥ 6 and CCI ≥ 3) are associated with an increase in mortality when adjusted by gender and age.

The most frequent comorbidities found in patients with solid tumors were cellulitis, acute renal failure, and hypertension for the CCI; meanwhile, pathologies such as pneumonia, septicaemia, and malnutrition were the most frequent for the PCI. An important observation in our study is the fact that the CCI was categorized as two comorbidity groups (high and low), while the PCI as 3 (absent, low, and high).

The presence of comorbidities in a disease increases the risk of complications, including death, impacting on clinical practice and hospital care costs. Williams et al. (20) showed that the incidence of comorbidities increases with age, and this is a subject of growing interest in the adult populations. Extermann (21) published that older cancer patients have an average of 3 comorbidities. Comorbidities have an impact on survival, disease progression and treatment in cancer patients, which proves that comorbidities negatively affect patient survival (22). It has been observed in adults that the older the patient is, the greater the comorbidity burden, and the most frequent comorbidities in adult cancer patients are cardiovascular diseases, infections, liver disorders and early malignancy (23, 24).

Currently, there are several indexes available to measure comorbidity conditions as mortality prognosis; however, none of these are designed for pediatric patients. In a retrospective study by Savic et al. (24) that included 233 adult patients with acute myeloid leukemia (AML) who were treated with intensive chemotherapy, the comorbidity index (comorbidity evaluation in adults-27-ACE-27) was able to predict survival in patients with several hematologic malignancies. Additionally, Wass et al. (25) confirmed in a study of 194 adult patients with AML, that comorbidities have a significant impact on the survival of patients and that a pre-treatment evaluation of comorbidities can help to identify those patients with unfavorable outcomes.

The comorbidity evaluation is considered of value to predict mortality in different groups of patients (26–29), and it may aid in decision- making, which can potentially lead to lower costs of care in pediatric patients with solid tumors.

CCI is the most commonly used comorbidity index validated in adult patients, but in our opinion, it does not appropriately represent the comorbidity burden found in pediatric patients. PCI based evaluation of comorbidity introduces 3 risk-group categories. While we acknowledge that this result is debatable, given that it is based on a subjective weighted score granted by an expert panel, it is significant that this method evaluates comorbidity burden in the early ages of life. This is done by taking into account the pathologies associated with mortality risk in hospitalized pediatric patients upon diagnosis.

We found similar results for both indexes, since for each comorbidity, the risk of mortality significantly increases by 15% (PCI) to 20% (CCI) when adjusting for age and gender. Age has a significant impact on the survival of patients, as reported in previous studies (30–32). Interestingly, in our study, the one-year increment in age was a statistically significant protective factor against mortality in the PCI, while gender showed no significance in either index.

The limitations for this study include aspects related to some retrospective data collection. We cannot exclude the possibility that the observed differences among categories in our analysis could be enhanced by differences between scoring methods for the PCI and CCI; however, we consider our findings valid because the evaluation of the comorbidity burden was strictly realized in both groups of the population studied. Sample size may also be considered a limitation, due to low prevalence of pediatric tumors; however, we consider it adequate and representative of patients managed at our tertiary pediatric hospital, who met the inclusion criteria during the study period. We acknowledge our findings require external replication in other pediatric populations.

## Conclusions

Comorbidities may affect treatment decisions, prognosis, and the evaluation of the quality of the patients' care. The comorbidity degrees of the PCI seem to facilitate better discrimination of the survival probability than the comorbidity degrees established in the CCI for pediatric cancer patients. The application of this index to the hospitalized pediatric population can be very useful for a baseline clinical evaluation that allows better decision-making during clinical follow-up.

## Data Availability

The datasets for this manuscript are not publicly available because of data confidentiality. Requests to access the datasets should be directed to mcastillejos@gmail.com.

## Author Contributions

LT-E and MC-L designed study. JD-R, CG-D, and MP-G performed follow-up of the patients and recorded the information. LT-E, AA-G, and MC-L analyzed the data, and wrote the manuscript. JG-M, LC-A and IT-R the manuscript and revised it. LT-E, MC-L, JD-R, CG-D, MP-G, AA-G, JG-M, IT-R, LC-A and JC-P reviewed the final version of the manuscript.

### Conflict of Interest Statement

The authors declare that the research was conducted in the absence of any commercial or financial relationships that could be construed as a potential conflict of interest.
